# Jejunal Proteins Secreted by *db/db* Mice or Insulin-Resistant Humans Impair the Insulin Signaling and Determine Insulin Resistance

**DOI:** 10.1371/journal.pone.0056258

**Published:** 2013-02-20

**Authors:** Serenella Salinari, Cyrille Debard, Alessandro Bertuzzi, Christine Durand, Paul Zimmet, Hubert Vidal, Geltrude Mingrone

**Affiliations:** 1 Department of Computer and System Science, University of Rome “Sapienza”, Rome, Italy; 2 Lyon 1 University, CarMeN Laboratory, INSERM U1060, Oullins, France; 3 Institute of Systems Analysis and Computer Science, National Research Council, Rome, Italy; 4 Baker IDI Heart and Diabetes Institute, Melbourne, Victoria, Australia; 5 Department of Internal Medicine, Catholic University, School of Medicine, Rome, Italy; University of Tor Vergata, Italy

## Abstract

**Background:**

Two recent studies demonstrated that bariatric surgery induced remission of type 2 diabetes very soon after surgery and far too early to be attributed to weight loss. In this study, we sought to explore the mechanism/s of this phenomenon by testing the effects of proteins from the duodenum-jejunum conditioned-medium (CM) of *db/db* or Swiss mice on glucose uptake *in vivo* in Swiss mice and *in vitro* in both Swiss mice soleus and L6 cells. We studied the effect of sera and CM proteins from insulin resistant (IR) and insulin-sensitive subjects on insulin signaling in human myoblasts.

**Methodology/Principal Findings:**

*db/db* proteins induced massive IR either *in vivo* or *in vitro*, while Swiss proteins did not. In L6 cells, only *db/db* proteins produced a noticeable increase in basal ^473^Ser-Akt phosphorylation, lack of GSK3β inhibition and a reduced basal ^389^Thr-p70-S6K1 phosphorylation. Human IR serum markedly increased basal ^473^Ser-Akt phosphorylation in a dose-dependent manner. Human CM IR proteins increased by about twofold both basal and insulin-stimulated ^473^Ser-Akt. Basal ^9^Ser-GSK3β phosphorylation was increased by IR subjects serum with a smaller potentiating effect of insulin.

**Conclusions:**

These findings show that jejunal proteins either from *db/db* mice or from insulin resistant subjects impair muscle insulin signaling, thus inducing insulin resistance.

## Introduction

Type 2 diabetes (T2D) is a heterogeneous disorder usually associated with insulin resistance and hyperinsulinemia leading to impaired glucose tolerance or frank diabetes as pancreatic insulin response declines [1].

Typically, obesity promotes insulin resistance with a compensatory increase in insulin production via increased β-cell mass [2], [3]. In obese T2D subjects, a prompt diabetes remission is observed after bariatric surgery [4], [5] and insulin sensitivity is restored [4], [6] along with the normalization of the first phase of insulin secretion [7]. Bariatric operations reroute food through the upper small intestine, possibly reducing the production of putative factor/s inducing insulin resistance and whose secretion is stimulated by nutrients. The mechanisms of T2D remission have been investigated in experimental animals. These suggest a pivotal role of the small intestine [8]. The bypass of duodenum and jejunum in Goto-Kakizaki (GK) rats, an animal model of non- obese T2D, was shown to control diabetes directly and not as a secondary effect of weight loss [9]. That food intake reduction is not implicated in the amelioration of glucose disposal was then demonstrated by the observation that GK rats which had undergone duodenal-jejunal bypass had a markedly better oral glucose tolerance compared to pair-fed sham-operated rats [9]. Duodenal-jejunal bypass surgery in rats normalized glucose disposal in streptozotocin-induced diabetes as well as in insulin deficient autoimmune type 1 diabetes [10].

Obese, diabetic C57BL/Ks *db/db* mice are an extensively studied genetic model of obesity and type 2 diabetes [11]. These animals show characteristics similar to human T2D including obesity and severe insulin resistance [12]. Brozinick et al. [13] have reported that despite the marked *in vivo* insulin resistance observed for the normal-glucose tolerant *db/db* mice during hyperinsulinemic clamps, their muscles are completely insulin responsive *in vitro*. On the basis of these findings, they suggested the presence of a humoral factor impairing the insulin action *in vivo* [13].

To test the hypothesis that the small intestine of *db/db* mice produces factors/hormones inducing insulin resistance, proteins enriched from the conditioned medium (CM) of *db/db* and Swiss duodenum-jejunum or of insulin resistant and insulin sensitive subjects were obtained. Their molecular cutoff was chosen in a range between 10 and 100 kDa, on the basis of a series of previous experiments. The biological activity of mouse CM proteins was assessed both *in vivo* in Swiss mice which underwent an intra-peritoneal insulin tolerance test, and *in vitro* in Swiss skeletal muscle tissue as well as in L6 cell cultures to measure insulin-mediated glucose uptake and insulin signaling. Furthermore, the effect on insulin signaling of serum or CM proteins from jejunum specimens obtained during abdominal surgery in insulin resistant and insulin sensitive human subjects was studied in human myotubes.

## Materials and Methods

### Experimental Animals

#### Animals

One-hundred fifty-eight (108M and 50F) Swiss mice 12–14 weeks old were from in-house breeding colonies. Eighty-one C57BL/6 (*db/db*) mice (31M and 50F) 12–14 weeks old were from Charles River Laboratories (Calco, Italy).

The protocol was approved by the Catholic University Animal Experimentation Ethics Committee in accordance with European guidelines for the use of animals.

#### Intestinal protein secretion

In order to enhance the secretion of intestinal factor/s inducing insulin resistance, fed mice were sacrificed by intraperitoneal injection with pentobarbital (12 mg/mouse, ip, Nembutal, Abbott Laboratories, Abbott Park, IL). Everted duodenum-jejunum sacs [14] were washed with saline and incubated in oxygenated (O_2_:CO_2_, 95∶5, v/v) Krebs-Henseleit solution (37°C, pH 7.4) for 1 h to isolate proteins secreted into the medium. Conditioned medium was lyophilized and stored at −80°C for protein purification.

#### Protein purification

Lyophilized powders were re-suspended in bidistilled water (∼10 mg/ml) and ultra-filtered through a hydrophilic 100 kDa cutoff membrane (Centripep, Amicon, Beverly) at 4°C. The fraction containing compounds <100 kDa was passed through a Centripep with membrane cutoff of 10 kDa. The material retained by the filter was removed with a pipette and dried.

Protein concentrations were determined with bicinchoninic acid (Pierce, Rockford, IL, USA), using bovine serum albumin as a concentration standard.

#### Intraperitoneal Insulin Tolerance Test (IPITT)

1 IU Actrapid insulin/kg bodyweight was injected into the peritoneum of Swiss mice after 16 h of fasting. Swiss mice were injected intravenously with saline (control) or proteins secreted from small intestine of *db/db* or Swiss mice 20 min before the IPITT. *t* = 0 is the starting time of intraperitoneal insulin injection. Blood samples were drawn from the orbital sinus and blood glucose measured by Accu-Chek blood glucose meter (Roche Diagnostics, Basel, Switzerland) at 0, 15, 30, 45, 60, and 90 min. Serum insulin was analyzed by radioimmunoassay (Linco Research, St. Charles, MO).

IPITT data were analyzed by adapting the minimal model [15], which is currently used to assess insulin sensitivity (*S_I_*, min^−1^⋅pM^−1^) and glucose effectiveness (*S_G_*, min^−1^) from intravenous or oral glucose tolerance-tests and has been validated in mice [16]. Minimal model equations were written in the following form
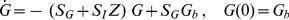
(1)

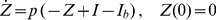
(2)where the overdot means *d/dt*, *G* is glucose concentration (basal value, *G_b_*), *I* insulin concentration (basal value, *I_b_*), *Z* a variable related to the insulin action, and *p* a rate constant (min^−1^) governing *Z* kinetics.

Insulin data, linearly interpolated, were assigned to *I* − *I_b_* in Eq. (2), and the model parameters *S_G_*, *S_I_*, *G_b_* and *p* were estimated by fitting glucose concentration data. Direct estimation of the population parameters was obtained by the NONMEM method [17].

#### Glucose transport in soleus muscle

Experiments were performed as reported elsewhere [18]. Briefly, Swiss soleus muscle was incubated for 20 min (glucose 5 mM, insulin 60 nM) in the absence (control) or in the presence of 10 µg/ml and 20 µg/ml of either db/db or Swiss CM proteins.

#### L6 cell culture

Skeletal L6 myoblasts were grown to 70–80% confluence in DMEM as described elsewhere [19], [20]. Cells were serum deprived for 2 h (glucose 25 mM) before treatment with various concentrations of insulin for 5 min. The rate of 2-DG uptake versus insulin concentration in L6 myoblasts was measured in the absence (control) or presence of 30 µg/ml Swiss or db/db CM.

2-deoxyglucose uptake data were fitted with a function of the insulin concentration, *I* :
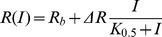
(3)where *R_b_* (pmol⋅min^−1^⋅well^−1^) is the rate of glucose uptake at zero insulin concentration, *ΔR* (pmol⋅ min^−1^⋅well^−1^) is the maximal insulin-stimulated increase in the rate of uptake, and *K*_0.5_ (nM) is the insulin concentration that yields the half-maximal increase.

#### Western blot analysis

L6 cells were harvested and lysed in ice-cold lysis buffer as previously described [21]. Akt phosphorylations were detected using anti-phospho-Ser^473^ and anti-phospho-Thr^308^ antibodies. For the characterization of the other signaling pathways in L6 cells, we used anti-^9^Ser-GSK3β and anti-^389^Thr-p70 S6K1. More details are given in the Supporting Information (Appendix S1).

### Humans

#### Demographic data

Seven obese and 3 normal weight subjects were enrolled. The study protocol was approved by the ethics committee of the Catholic University, School of Medicine, Rome, Italy. All the subjects signed two written informed consents, one prior to the study and the other before surgery.

The seven IR subjects had an mean age of 41.7±4.6 years and a body mass index (BMI) of 43.24±1.91 kg/m^2^. The three normal weight subjects were 41.0±3.6 (mean ± s.d.) years old with a BMI of 22.28±1.85 kg/m^2^.

#### Insulin sensitivity assessment

Insulin sensitivity was assessed by the euglycemic hyperinsulinemic clamp [22]. Insulin sensitivity was determined during the last 40 min of the clamp by computing the whole-body glucose uptake (M, µmol⋅min^–1^⋅kg_bw_^–1^) during the steady-state euglycemic hyperinsulinemia.

#### Surgery

The obese, insulin resistant subjects underwent Roux-en-Y gastric bypass after an overnight fast. Specimens of the jejunum of circa 500 mg were obtained during the operation and immediately placed in the oxygenated incubation medium. After an overnight fast, the three insulin sensitive subjects underwent elective small bowel resection for stenosis of the terminal ileum in inactive Crohn’s disease, taking care during the operation to obtain the jejunal specimens (ca. 500 mg) at a distance of at least 15 cm from the diseased portion of the small intestine.

#### Human skeletal muscle cell culture and Western blot analysis

Human myoblasts were plated on 6-well plates coated with collagen I and grown in Ham’s F10 medium supplemented with 2% FCS, 2% Ultroser G, and 1% antibiotics. Differentiation was induced in DMEM (1 g/l glucose) without serum and polynucleated myotubes were obtained after 4 days [23]. Myotubes were treated for 15 min with serum from IR patients or with conditioned medium proteins (25 µg/ml) from IR or insulin sensitive patients in the presence or absence of 100 nM of human recombinant insulin.

The western blot conditions are described in the Supporting Information (Appendix S1).

#### Statistical analysis

Data are presented as mean ± s.d. unless otherwise indicated. ANOVA test for repeated measurements followed by Tukey-test was used for intergroup comparisons. Two-sided *P*<0.05 was significant.

## Results

### Experimental Animal Data

#### Intraperitoneal insulin tolerance test

**Figure 1:**
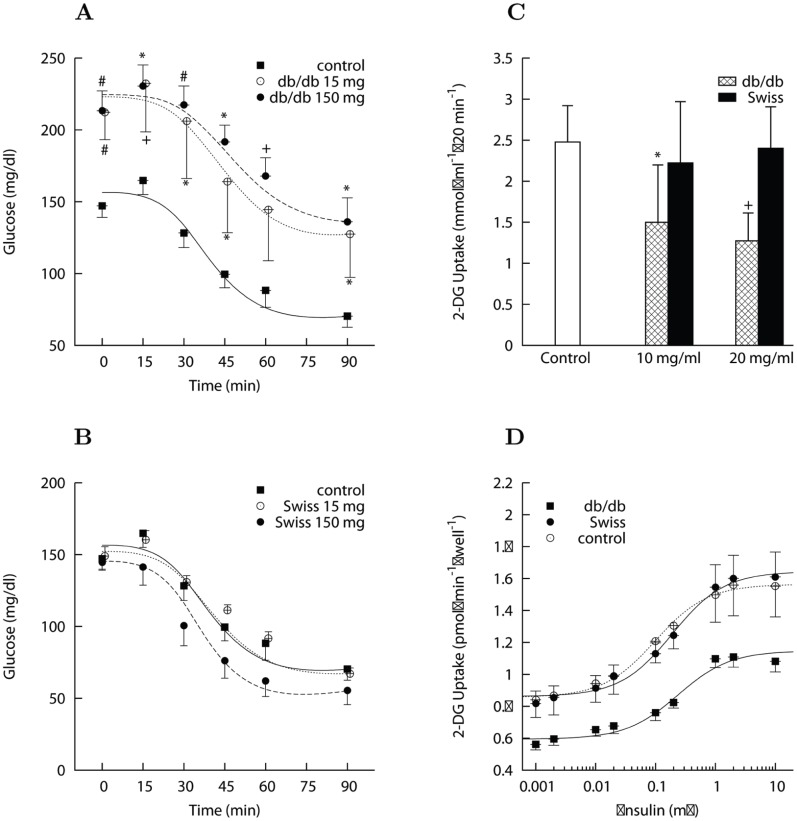
Intraperitoneal insulin tolerance test on Swiss mice and effect of *db/db* and Swiss conditioned medium (CM) proteins on the *in vitro* glucose uptake. *Panel A*: Glucose concentration profiles during the IPITT in controls (*n* = 23) and mice injected with 15 (*n* = 10) or 150 µg (*n* = 11) of *db/db* CM proteins. Values are mean ± s.e.m. *P<0.05, ^+^ *P*<0.01, and ^#^ *P*<0.001, *db/db* 15 or 150 µg vs. control. The lines are the fitting curves obtained by the model of Eqs. (1)-(2) in Methods with the parameter values reported in Table 1. *Panel B*: Glucose concentration profiles during the IPITT (mean ± s.e.m.) in controls (*n* = 23) and mice injected with 15 (*n* = 11) or 150 µg (*n* = 7) of Swiss CM proteins. *Panel C*: Transport of 2-deoxyglucose (2-DG) in isolated soleus muscle. Values are mean ± s.d. of *n* = 7 determinations of 2-DG uptake expressed as µmol per ml intracellular water per 20 min. *P<0.02, *db/db* 10 µg/ml CM proteins vs. control; ^+^ *P*<0.0005, *db/db* 20 µg/ml CM proteins vs. control. *Panel D*: Rate of 2-DG uptake versus insulin concentration in L6 myoblasts. Insulin mediated glucose uptake was determined in cells exposed to 30 µg/ml of *db/db* or Swiss CM proteins vs. control. Data points are mean ± s.d. of *n* = 7 determinations for each insulin concentration expressed as pmol per min per well. *P*<0.0001 for 2-DG uptake reduction with *db/db* CM proteins vs. control and Swiss CM at all insulin concentrations. Fitting curves are given by Eq. 3 in Methods with the parameter values reported in Table 2.

The effect on insulin sensitivity of saline (control) and CM proteins from *db/db* or Swiss mice injected in Swiss mice was evaluated by the intraperitoneal insulin test (IPITT) and minimal model analysis (Fig. 1, A and B). Amounts of proteins injected were 15 or 150 µg, which corresponded to circulating levels of 2.1 or, respectively, 21 µg/ml assuming that the distribution volume of these proteins equals that of glucose (7.1 ml, see [16]). The overall profile of glucose concentration was significantly increased in animals injected with either 15 or 150 µg proteins from *db/db* mice, compared with animals injected with saline (Fig. 1A), strongly suggesting that *db/db* proteins induce insulin resistance in control Swiss mice. In contrast, glucose concentration profiles in mice injected with Swiss proteins did not differ from those of controls (Fig. 1B). The estimates of minimal model parameters (Eqs. (1)-(2) in Methods) demonstrate that the insulin sensitivity, *S_I_*, that accounts for cumulative insulin action in liver and peripheral tissues, was significantly decreased in mice injected with *db/db* proteins (ca. 40% with both 15 and 150 µg) (Table 1). The glucose effectiveness, *S_G_*, representing the capacity of glucose to regulate its own disposal, was also significantly reduced in the presence of *db/db* proteins. By contrast, the parameters from mice treated with Swiss proteins did not differ significantly from those of controls.

**Table 1:** Estimates of parameters (mean ± s.d. over the population) of Eqs. (1)-(2) used to fit IPITT data.

CM proteins	Control n = 23	Swiss 15 µg n = 11	Swiss 150 µg n = 7	*db/db* 15 µg n = 10	*db/db* 150 µg n = 11
*S_G_* (min^−1^)×10^2^	2.61±0.43	2.95±1.04	2.15±1.63	2.20±0.34	1.01±0.75# *°
*S_I_* (min^−1^⋅pM^−1^)×10^5^	8.45±1.95	8.73±4.55	9.51±3.50	4.50±1.52?×	5.42±1.22&°
*G_b_* (mg⋅dl^−1^)	156.9±38.6	152.2±21.6	145.4±12.7	223.0±59.9+	224.5±46.0+
*p* (min^−1^)×10^2^	1.77±1.22	1.47±0.40	2.32±1.56	3.01±1.32	2.93±1.43

# *P*<0.001, *db/db* 150 µg vs. control and Swiss 15 µg;

+ *P*<0.005, *db/db* 15 or 150 µg vs. Swiss 15 or 150 µg;

?*P*<0.005, *db/db* 15 µg vs. control;

× *P*<0.01, *db/db* 15 µg vs. Swiss 15 and 150 µg;

* *P*<0.05, *db/db* 15 vs. *db/db* 150 µg;

°*P*<0.05, *db/db* 150 µg vs. Swiss 150 µg;

& *P*<0.05, *db/db* 150 µg vs. control and Swiss 15 µg.

#### Glucose uptake in soleus muscle

To test the effect of CM proteins on glucose uptake in muscle, 2-deoxyglucose transport in response to a constant insulin concentration of 60 nM was measured *in vitro* in the isolated soleus muscle of Swiss mice (Fig. 1C). Glucose transport – equal in control to 2.48±0.44 (mean ± s.d.) µmol of 2-deoxyglucose (2-DG) per ml intracellular water over 20 min – was reduced by 39.5% when muscle samples were exposed to 10 µg/ml of *db/db* proteins and 49.7% when exposed to 20 µg/ml. By contrast, in the presence of the same concentrations of Swiss proteins, the rate of glucose transport underwent a smaller, not significant reduction.

#### Glucose uptake in L6 cells

The effects of the exposure to proteins from *db/db* mice on the insulin-stimulated glucose uptake in L6 myoblasts (Fig. 1D) were consistent with those observed during the IPITT and the glucose transport experiments in the isolated soleus muscle. After preincubation with 30 µg/ml *db/db* proteins, the 2-DG rate of uptake, expressed as pmol⋅min^−1^⋅well^−1^, was significantly reduced with respect to control at all insulin concentrations from 1 nM to 10 µM. In contrast, the response to insulin under the exposure to the same concentration of Swiss proteins was not statistically different from that observed in response to saline.

Table 2 reports the parameters *R_b_*, *ΔR*, and *K*_0.5_ of Eq. (3) in Methods, estimated by fitting the glucose uptake data. The ratio *ΔR*/*R_b_* was close to the unity in all cases because, as also shown by the raw data, the maximal 2-DG uptake was about twofold the basal uptake either in control or in the presence of the intestinal proteins. However, the insulin-independent rate of uptake, *R_b_*, which is possibly related to the activity of GLUT1, was found to be largely decreased only in cells exposed to *db/db* proteins.

**Table 2:** Estimates of parameters (estimate ± s.d. of the estimate) of Eq. (3) used to fit the rate of deoxyglucose uptake in L6 cells.

Secreted proteins	Control	Swiss	*db/db*
*R_b_* (pmol⋅min^−1^⋅well^−1^)	0.855±0.112	0.847±0.077	0.593±0.033* &
*ΔR* (pmol⋅min^−1^⋅well^−1^)	0.713±0.130	0.782±0.150	0.555±0.055 #
*K*_0.5_ (nM)	100.7±34.7	190.8±120.2	240.5±52.1+

* *P*<0.00005, *db/db* vs. control;

+ *P*<0.02, *db/db* vs. control;

& *P*<0.00005, *db/db* vs. Swiss;

# *P*<0.01, *db/db* vs. Swiss.

A measure of the sensitivity to insulin of glucose uptake was obtained by computing from Eq. (3) the derivative of the rate of uptake *R* with respect to *I* at *I = *0, which gives (*dR/dI* )|*_I = _*_0_ = *ΔR*/*K*_0.5_. The values of this ratio (µl⋅min^−1^⋅well^−1^) were 7.1±1.8 in control, 4.1±1.8 in the presence of Swiss proteins and 2.3±0.3 in the presence of *db/db* proteins, respectively, showing that insulin sensitivity was significantly decreased (*P*<0.0002) in the L6 cells exposed to *db/db* proteins.

We found that the average reduction in glucose uptake (37.2%) by the L6 cells in the presence of *db/db* proteins at 60 nM insulin concentration as computed by Eq. (3) was in the same order of the decrease found in the isolated soleus muscle.

#### Effects of mice CM proteins on insulin signaling in L6 cells

**Figure 2:**
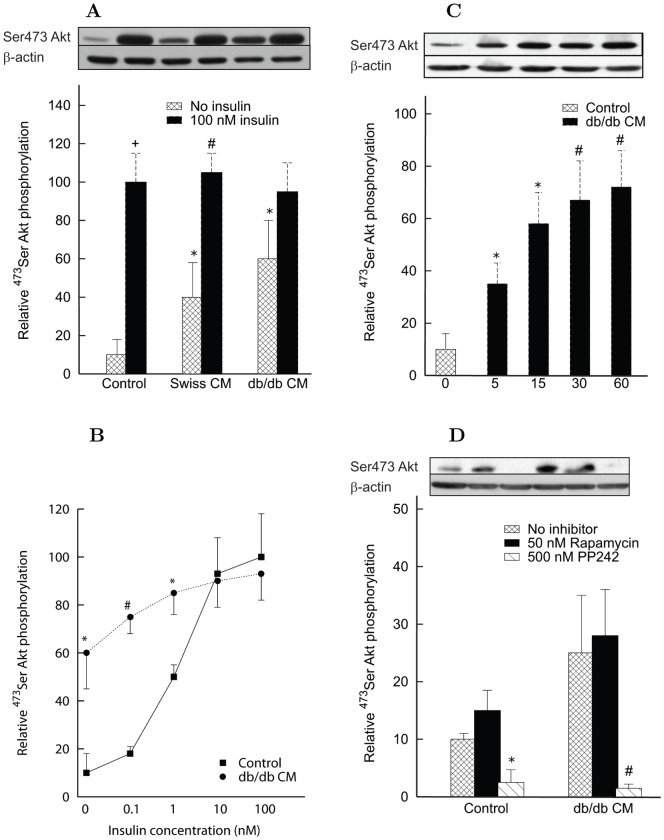
Effect of conditioned medium (CM) on ^473^Ser Akt phosphorylation in differentiated L6 myotubes. Each bar is the mean of experiments in triplicate with 3 to 5 different preparations of CM. Data are normalized by β-actin amount and expressed as fold change versus control condition (control at 100 nM insulin set at 100). Data are mean ± s.d. *Panel A*: 15 min incubation of L6 myotubes with 25 µg/ml of CM proteins increased ^473^Ser Akt phosphorylation level in the absence of insulin, with an effect that tended to be stronger of medium prepared from *db/db* (*P<0.05 vs. control) than from Swiss mice. The stimulatory effect of 100 nM insulin in control (^+^ *P*<0.01 vs. no insulin) was preserved in the presence of Swiss CM (^#^ *P*<0.05) but did not reach significance in the presence of *db/db* CM. *Panel B*: Dose-response profile of insulin action on ^473^Ser Akt phosphorylation vs. insulin concentration (nM) in logarithmic scale after 15 min co-incubation in the absence (squares) or presence (circles) of 25 µg/ml *db/db* CM proteins (^#^ *P*<0.01 and ^*^ *P*<0.05 vs. control). *Panel C*: Basal (no insulin) ^473^Ser Akt phosphorylation determined after different incubation times with 25 µg/ml *db/db* CM proteins. An increased ^473^Ser Akt phosphorylation was already observed after 5 min of incubation and approached saturation at 15–30 min (^*^ *P*<0.05 and ^#^ *P*<0.01 vs. control). *Panel D*: Effect of mTORC inhibitors on basal ^473^Ser Akt phosphorylation. L6 myotubes were treated with Rapamycin or PP242 for 1 hour before 15 min incubation with 25 µg/ml CM proteins. The stimulatory effect of *db/db* proteins was inhibited by PP242 in both control (^*^ *P*<0.05 vs. no inhibitor) and *db/db* CM treated cells (^#^ *P*<0.01 vs. no inhibitor).

CM proteins at a concentration of 25 µg/ml increased ^473^Ser Akt phosphorylation in the absence of insulin, with a tendency toward a stronger effect of medium prepared from *db/db* than from Swiss mice (Fig. 2A). This effect was very rapid, already observed after 5 min of incubation, and approached saturation at 15–30 min (Fig. 2C). Insulin was still able to increase ^473^Ser Akt phosphorylation in the presence of CM proteins from *db/db* mice, but the amplitude of the maximal increase over basal was markedly reduced (Fig. 2A). Accordingly, the sigmoidal profile of the dose-response curve of insulin action on ^473^Ser Akt phosphorylation was lost in the presence of *db/db* proteins (Fig. 2B). Rapamycin, a classical inhibitor of mammalian target of Rapamycin complex 1 (mTORC1), did not counteract the effect of CM proteins from *db/db* mice on ^473^Ser Akt phosphorylation, while the global mTOR inhibitor PP242 completely abolished this phosphorylation, particularly in cells incubated with *db/db* CM proteins (Fig. 2D). This result suggests that CM proteins probably increased ^473^Ser Akt phosphorylation in muscle cells through stimulation of mTORC2 complex.

Insulin stimulated ^308^Thr Akt phosphorylation had a tendency toward a decrease (*P* = 0.08) in the presence of *db/db* but not Swiss CM proteins (Fig. S1A), indicating that *db/db* CM proteins tend to inhibit phosphoinositide-dependent kinase 1 (PDK1) activity. Accordingly, the dose-response curve of insulin action on ^308^Thr Akt phosphorylation was reduced in the presence of *db/db* proteins (Fig. S1B).

**Figure 3:**
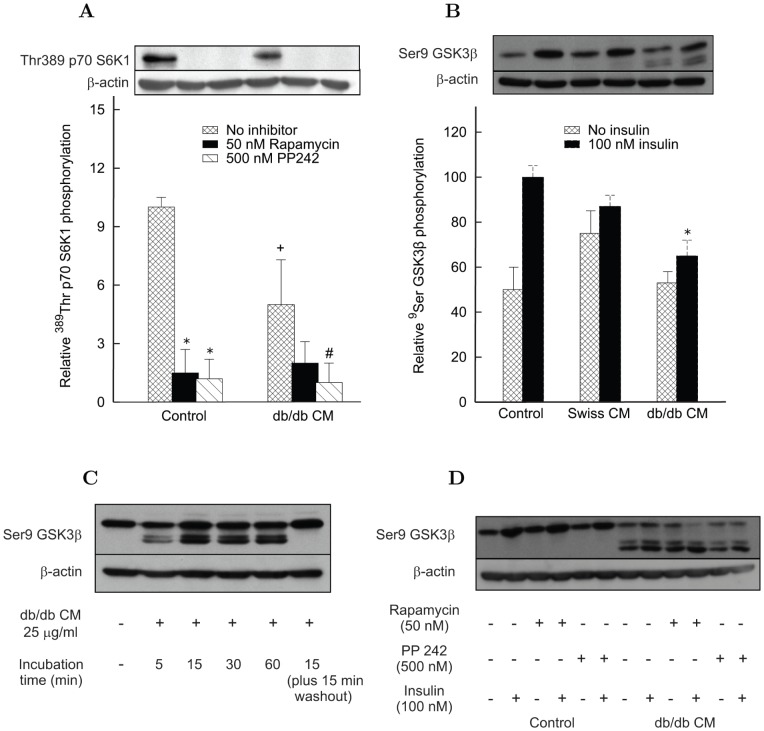
Effect of conditioned medium (CM) on p70 S6kinase and GSK3β phosphorylation in differentiated L6 myotubes. Each bar is the mean of experiments in triplicate with 3 to 5 different preparations of CM. Data are normalized by β-actin amount and expressed as fold change versus control condition (no insulin nor CM) set at 10. Data are mean ± s.d. *Panel A*: ^389^Thr p70 S6K1 phosphorylation. L6 myotubes were treated with Rapamycin or PP242 for 1 hour before 15 min incubation with 25 µg/ml of *db/db* CM. *db/db* CM proteins inhibited the basal ^389^Thr p70 S6K1 phosphorylation (^+^ *P*<0.05 vs. control no inhibitors). PP242 further reduced the phosphorylation level in both control (^*^ *P*<0.01, Rapamycin and PP242 vs. no inhibitor) and *db/db* CM (^#^ *P*<0.05 vs. no inhibitor). *Panel B*: ^9^Ser GSK3β phosphorylation after 15 min co-incubation with 25 µg/ml CM with or without insulin. *db/db* CM proteins inhibited the insulin-stimulated increase of ^9^Ser GSK3β phosphorylation (^*^ *P*<0.05 vs. insulin-induced phosphorylation in control set at 100). Lower molecular weight bands in the blot suggest proteolysis of phosphorylated form of GSK3β upon *db/db* CM treatment. *Panel C*: Time course of the effect of 25 µg/ml *db/db* CM on ^9^Ser GSK3β phosphorylation and proteolysis. The *db/db*-derived conditioned medium did not significantly affect basal phosphorylation of GSK3β on ^9^Ser, while it induced a rapid degradation of GSK3β, measurable since 5 min of incubation. After 15 min of pretreatment with *db/db* CM, a washout for 15 min with medium replacement was able to fully correct the alterations induced by the *db/db* CM. *Panel D*: Pretreatment of L6 myotubes with Rapamycin or PP242 for 1 hour before incubation with 25 µg/ml CM from *db/db* for 15 min in the absence or presence of 100 nM insulin. mTOR inhibitors had no effect on proteolysis of phosphorylated GSK3β induced by *db/db* CM proteins either in the presence or absence of insulin.

To further confirm the implication of mTOR signaling in the effect of CM proteins, we measured the phosphorylation of S6Kinase in L6 cells. In control conditions, both Rapamycin and PP242 abolished the basal ^389^Thr phosphorylation of p70 S6K1 (Fig. 3A), consistent with the role of mTORC1 in mediating ^389^Thr phosphorylation in its downstream substrate S6K1. Interestingly, *db/db* CM proteins in the absence of inhibitors reduced by about 50% (*P*<0.05) the basal ^389^Thr phosphorylation of p70 S6K1 (Fig. 3A). Addition of the mTOR inhibitors further suppressed the phosphorylation of S6K1 in the presence of the *db/db* CM, suggesting that CM proteins could negatively control mTORC1 activity (Fig. 3A) while concomitantly activating mTORC2 (Fig. 2D).

Glycogen synthase kinase 3 (GSK3) is a well-known downstream substrate of Akt. In human muscle cells, insulin stimulates GSK3β phosphorylation on the ^9^Ser residue, leading to inhibition of its activity [24]. In L6 cells, incubation with *db/db* CM proteins led to a marked inhibition of insulin-stimulated increase of ^9^Ser GSK3β phosphorylation (Fig. 3B). Furthermore, lower molecular weight bands appeared on the blot, suggesting proteolysis of the phosphorylated form of GSK3β especially upon *db/db* CM protein treatment (Fig. 3B). Actually, a rapid proteolysis of ^9^Ser phosphorylated GSK3β was observed, readily after 5 min incubation, in the presence of *db/db* CM, and this effect was fully reversible on 15 min washout with fresh culture medium (Fig. 3C). Finally, mTOR inhibitors had no effect on the proteolysis of phosphorylated GSK3β induced by the *db/db* CM proteins, either in the presence or absence of insulin (Fig. 3D).

### Human Data

#### Euglycemic-hyperinsulinemic clamp

The insulin mediated glucose disposal (M) in the IR subjects was much lower than in Crohn’s patients. The M value of the former group was 16.4±2.8 µmol⋅min^–1^⋅kg_bw_^–1^ with a steady state plasma insulin concentration of 460.3±89.7 pmol/l, whereas in the in Crohn’s patients the M value was 47.4±3.6 µmol⋅min^–1^⋅kg_bw_^–1^ with a plasma insulin concentration at the steady state of 420.0±26.2 pmol/l.

#### Effects of human serum and CM proteins on insulin signaling in human myotubes

**Figure 4:**
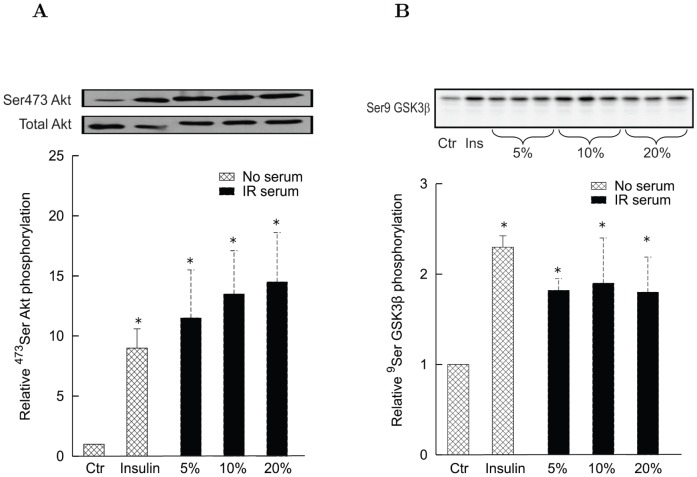
Effect of serum from IR subjects (15 min) on Akt and GSK3β phosphorylation in differentiated human myotubes. Each bar is the mean of the results with sera from 7 IR subjects. Experiments were performed in triplicate for each individual. Ctr denotes controls. *Panel A*: Basal ^473^Ser Akt phosphorylation in the presence of 100 nM insulin or of different serum concentrations (5%, 10% and 20%) from IR subjects. Blots are representative western blots of ^9^Ser Akt phosphorylation and total Akt amounts. Data (mean ± s.d.) are expressed as fold change versus control condition (no insulin nor serum) set at 1 (^*^ *P*<0.05 vs. control). *Panel B*: Basal ^9^Ser GSK3β phosphorylation in the presence of 100 nM insulin or of different serum concentrations (5%, 10% and 20%) from IR subjects. The blot is a representative western blots of ^9^Ser GSK3β phosphorylation. Data (mean ± s.d.) are expressed as fold change versus control condition (no insulin nor serum) set at 1 (^*^ *P*<0.05 vs. control).

To assess whether the findings in L6 cells are replicated in humans, Akt and GSK3β phosphorylation was measured in human myotubes treated with serum or CM proteins from insulin sensitive and insulin resistant subjects. Incubation of human myotubes with 5, 10 or 20% serum from IR subjects induced a rapid (15 min) and dose dependent increase of basal (no insulin)^ 473^Ser Akt phosphorylation, which attained a level comparable with that induced by 100 nM insulin (Fig. 4A). Similarly, serum induced ^9^Ser GSK3β phosphorylation with levels comparable with that of insulin (Fig. 4B). By contrast, no significant changes were obtained with sera from insulin sensitive subjects (data not shown).

**Figure 5:**
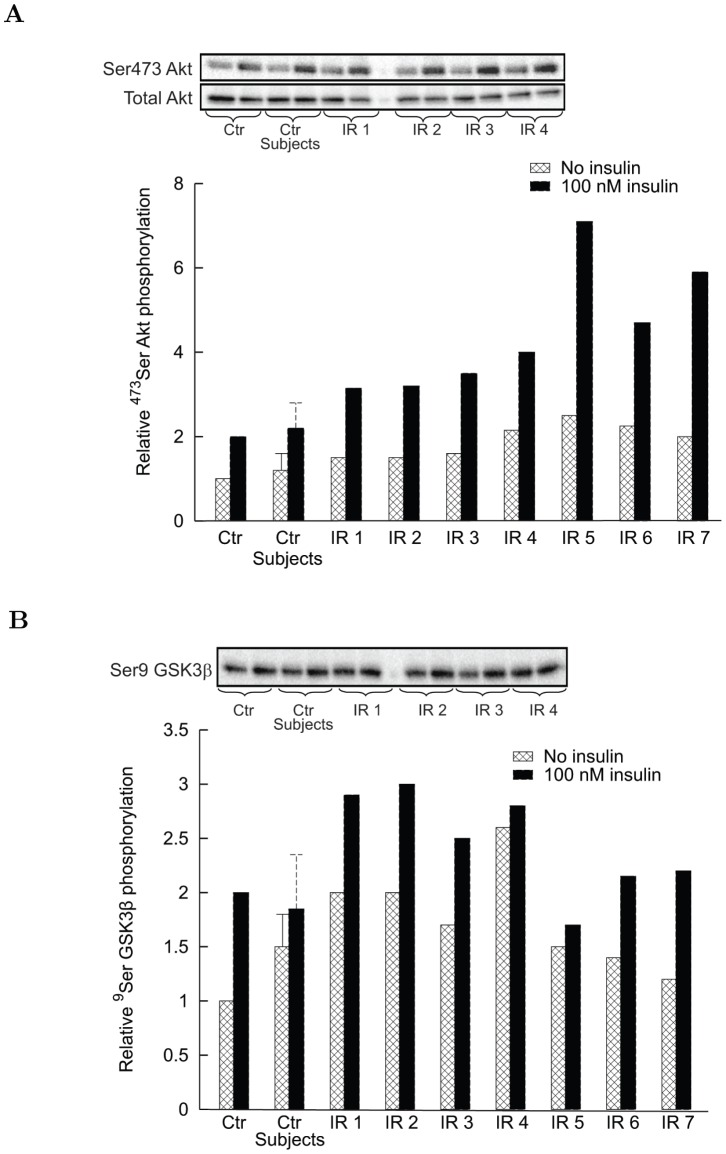
Effect of conditioned medium (CM) from insulin sensitive subjects and IR subjects on Akt and GSK3β phosphorylation in differentiated human myotubes. Control denotes phosphorylation in the absence of CM, with phosphorylation level in the absence of insulin set at 1. Data are reported as mean ± s.d. for control subjects (Crohn’s patients denoted as control subjects), while individual values are reported for each IR subject. Each bar is the mean of experiments in triplicate. *Panel A*: 15 min incubation of human myotubes from insulin sensitive subjects with 25 µg/ml of CM proteins increased ^473^Ser Akt phosphorylation level in the absence of insulin, with a stronger effect of medium prepared from IR subjects than from insulin sensitive subjects (*P*<0.05 vs. control subjects). The stimulatory effect of 100 nM insulin in control was increased in the presence of CM from IR subjects (*P*<0.01 vs. control subjects). Blots are representative western blots of ^473^Ser Akt phosphorylation and total Akt amounts. *Panel B*: 15 min incubation of human myotubes from insulin sensitive subjects with 25 µg/ml of CM proteins increased ^9^Ser GSK3β phosphorylation level in the absence of insulin, with a stronger effect of medium prepared from IR subjects than from insulin sensitive subjects (*P*<0.05 vs. control subjects). The incremental stimulatory effect of 100 nM insulin over basal was reduced mainly in the presence of CM from IR subjects. Blot is a representative western blot of ^9^Ser GSK3β phosphorylation.

The intestinal CM proteins from insulin resistant subjects almost doubled the basal level of ^473^Ser Akt phosphorylation with respect to insulin sensitive controls, but also its insulin stimulated level was markedly increased (Fig. 5A). The degree of insulin sensitivity (M in µmol⋅min^–1^⋅kg_bw_^–1^ normalized by the steady state insulin, I, in pM) measured by the euglycemic clamp inversely correlated with the relative amount of ^473^Ser Akt during insulin stimulation. The linear regression equation is: M/I = 0.128 − 0.018 ^473^Ser Akt (R^2^ = 0.613, *P = *0.007).

Basal ^9^Ser GSK3β phosphorylation had a tendency to increase in the presence of CM proteins from IR subjects compared with insulin sensitive subjects, while the differential increase of phosphorylation under insulin stimulation was pretty low (Fig. 5B).

## Discussion

Two recent studies demonstrated that bariatric surgery induced remission of T2D and significant improvement in metabolic control of the diabetes over and above medical therapy [5], [25]. The improvement in glycemia occurs very soon after the surgery and far too early to be attributed to weight loss. In this study, we sought to explore the mechanism/s of this phenomenon. Jejunal conditioned medium proteins from both diabetic, insulin resistant animals and insulin resistant humans impaired insulin signaling in skeletal muscle cell cultures. A similar effect was obtained with the human serum from insulin resistant subjects, suggesting that circulating (and thus by definition endocrine) factors are inducing insulin resistance.

An *in vivo* and *in vitro* state of insulin resistance was reproduced in the presence of proteins secreted by the duodenal-jejunal mucosa of *db/db* mice. Contrary to the conditioned medium proteins from Swiss mice, those secreted by the *db/db* mice small intestine induced insulin resistance when injected in normal Swiss mice or when added to the incubation medium of skeletal muscle preparations *in vitro*. Also the experiments with the L6 cells were congruent, as shown by ∼65% and ∼30% reduction of insulin-dependent (*ΔR*/*K*_0.5_) and insulin-independent (*R_b_*) glucose uptake in the presence of *db/db* proteins, respectively. Notably, Swiss mice proteins failed to decrease glucose uptake either *in vivo* or *in vitro*.

Our study demonstrates that the molecular mechanism through which proteins secreted by the duodenal-jejunal mucosa of *db/db* mice induce insulin resistance could be mediated by interference with intracellular signaling pathways. We found that *db/db* proteins robustly induced the phosphorylation of the Akt ^473^serine residue at zero insulin, leading to a prompt saturation of the insulin dose-response curve. Moreover, *db/db* CM proteins tended to reduce the insulin-induced phosphorylation of Akt on ^308^Thr residue. Thus, 100 nM insulin treated L6 cells in the presence of *db/db* CM exhibited maximal Akt phosphorylation at ^473^Ser residue but about 50% phosphorylation at ^308^Thr residue, so full Akt activation could not be attained, consistent with the observed reduction of 2DG uptake in the soleus muscle and L6 cells. CM *db/db* also determined a marked proteolysis of GSK3β phosphorylated form and inhibited the basal activation of the p70 S6kinase.

Interestingly, when compared with insulin sensitive individuals, both serum and intestinal CM proteins from insulin resistant subjects determined a higher basal ^473^Ser Akt and ^9^Ser GSK3β phosphorylation in human myotubes. A tendency toward an increased basal phosphorylation of ^9^Ser GSK3β was also observed in the presence of CM from insulin sensitive subjects. Furthermore, the incremental ^9^Ser GSK3β phosphorylation over basal in the presence of insulin was reduced in IR subjects. Overall, these data suggest that serum and conditioned medium contain factor/s, which are likely in a larger amount in the insulin resistant than in insulin sensitive subjects, that induce insulin resistance. We observe that we could not compare insulin resistant with healthy control subjects because it is near impossible to obtain sufficient duodenal/jejunal mucosa during endoscopy to provide sufficient intestinal secreted proteins for testing in human myoblasts. Our controls were in an inactive phase of their disease with stenosis of the ileum which required elective surgery. We cannot exclude a certain degree of general inflammation, and thus they could not be considered as healthy subjects. However these patients were still insulin sensitive according to a previous observation [26] and as assessed by the euglycemic clamp.

The Akt phosphorylation of ^473^Ser residue is a target of the mTOR complex 2 [27], while the Akt phosphorylation at ^308^Thr is operated by PDK1, this last step being essential for the full Akt catalytic activity [28], [29]. Fraenkel et al. [30] showed that, in the fasting state, basal ^473^Ser Akt phosphorylation was higher in the skeletal muscle of diabetic than in normoglycemic *Psammomys obesus* (*P. obesus*), while there was a net reduction of the stimulation by insulin in agreement with the high insulin resistance state of these diabetic animals. Furthermore, it was found that Akt directly mediates Ser/Thr phosphorylation of the insulin receptor substrate 1 (IRS-1), resulting in a negative feedback loop that reduces insulin action [31]. Interestingly, this effect was inhibited by Rapamycin [31].

Akt phosphorylates ^9^Ser in GSK3β with subsequent inhibition of the enzymatic function of GSK [32]. It was recently demonstrated that GSK activity can also be regulated by calpain-induced proteolysis of its N terminus, which gives way to a short-lived constitutively active form of the enzyme [33]. Our data suggest that similar mechanisms could occur in the presence of small intestine *db/db* CM. If the products of GSK3β degradation induced by *db/db* CM proteins are active, they might contribute to enhance the recognition of GSK substrates. In addition to the well-known effect on glycogen synthase activity [34], [35], GSK3 has been implicated in the phosphorylation of the IRS-1 on serine residues with a consequent impairment of insulin signaling [36], [37]. Furthermore, GSK3 overexpression was found in peripheral tissues in a variety of diabetic animals and also in humans [38]–[42]. It has also been shown that Rapamycin markedly decreased GSK3 phosphorylation in muscle of normoglycemic and diabetic *P. obesus*, indicating increased GSK3β activity [30], while diabetes was reversed in obese diabetic mice treated with GSK3 inhibitors [43], [44].

The mammalian target of rapamycin (mTOR) exists in two forms, mTORC1 and mTORC2. mTORC1 regulates protein synthesis by S6K1 and the eukaryotic initiation factor 4E-binding protein 1 at ribosomal level [45], while mTORC2 phosphorylates Akt at ^473^Ser. Rapamycin is an mTORC1 inhibitor, which acts specifically on S6K1, whereas the ATP-competitive inhibitor PP242 completely blocks both mTORCs [46]. We found that the effect of *db/db* CM proteins on Akt ^473^Ser phosphorylation was fully prevented by PP242, but not by Rapamycin, indicating a preferential role of mTORC2 in the response to CM proteins in L6 cells. Furthermore, inhibition of p70S6K1 phosphorylation suggested a negative modulation of mTORC1 inhibitors.

**Figure 6:**
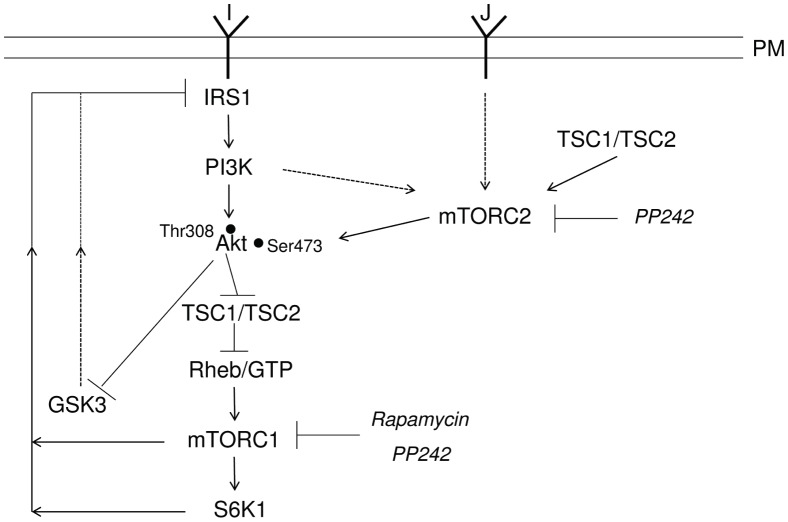
Interaction between IRS1−PI3K−Akt signaling pathway and mTOR. Upon insulin binding insulin receptor activates, through insulin receptor substrate 1 (IRS1), phosphatidylinositol-3-kinase (PI3K), which results in Akt phosphorylation at ^308^Thr residue via Phosphatidylinositol 4,5-bisphosphate (PIP2) to Phosphatidylinositol 3,4,5-bisphosphate (PIP3) conversion followed by Akt and PDK1 recruitment to plasma membrane (not shown). A factor J present in duodenal-jejunal conditioned medium activates, possibly via the tuberous sclerosis complex TSC1-TSC2, the mammlian target of rapamycin complex 2 (mTORC2). mTORC2 also appears to be regulated by the PI3K pathway and phosphorylates Akt at ^473^Ser residue. Through TSC1-TSC2 and the GTPase Rheb, Akt activates mTORC1 and its direct substrate S6K1. Akt also inhibits GSK3. S6K1, mTORC1 and GSK3 phosphorylate serine residues on IRS1, thus attenuating insulin signalling. Hypothetical signalling is denoted by dotted lines.

Taken together, these results support a possible mechanism of action of the *db/db* or IR subjects CM on insulin signaling and action in skeletal muscle cells by which proteins produced by small intestine could act via activation of mTORC2 while inhibiting mTORC1. Activation of mTORC2 may be regulated by activation of TSC1/TSC2 complex, which directly binds to mTORC2 while inhibiting Rheb and thus mTORC1 [47]. So, the increased ^473^Ser Akt phosphorylation is accompanied by decrease in basal (no insulin) p70 S6K1 ^389^Thr phosphorylation. Accordingly, Rapamycin and PP242 inhibit p70 S6K1 ^389^Thr phosphorylation (as also observed in the absence of *db/db* CM). This is accompanied by the lack of inhibition of GSK3 due to the proteolysis of its phosphorylated form, and may in turn lead to the phosphorylation of IRS-1 on serine/threonine residues that determines inhibition of insulin signaling by reducing IRS-1 tyrosine phosphorylation with consequent insulin resistance [48] (Fig. 6).

Our finding that *db/db* CM promotes Akt ^473^Ser phosphorylation in L6 cells, likely via mTORC2 or TSC activation and Akt recruitment to plasma membrane, may provide a new insight on the upstream regulation of mTORC2 [49]. Finally, the reversibility of CM proteins action after washout suggests that these proteins may act through the activation of a membrane receptor. We conclude that, although some difference in the effect of mice and human CM proteins on the insulin signaling does exist, the mechanism of action, as summarized in Figure 6, likely involves the mTOR pathway.

The strength of the present study is the demonstration that the small intestine of insulin resistant humans and mice secrete a protein factor/s inducing insulin resistance by impairing the insulin signaling.

The limitations of our investigation are that insulin sensitivity in mice was assessed by the IPITT instead of a more sophisticated euglycemic hyperinsulinemic clamp and that the insulin signaling pathway was studied in two different cellular lines, the L6 cells to test the proteins secreted by the mice intestine and human myoblasts to test the proteins secreted by the human intestine. Concerning the first point we note that the steady state conditions of plasma concentration, required by the clamp, could not be assured for the jejunal proteins, whose kinetics is not known. Moreover, the minimal model analysis also provides the estimate of the glucose effectiveness that has been found decreased after the *in vivo* injection of *db/db* proteins.

As for the second point, it is well accepted that insulin action is comparable in L6 cells and primary muscle cells. Because the use of human primary muscle cells requires biopsies and time-consuming expansion and differentiation, we restricted the use of these cells to the study of the human secreted proteins. Actually, we found similar results by using the secreted proteins from *db/db* mice and insulin resistant humans, although the proteolytic degradation of phopsphorylated GSK3, observed in the rodent study, was not present in the human study.

Ideally, the best model to investigate the role of the small intestine in inducing insulin resistance would be that of studying the same insulin resistant subjects and animals before and after bariatric surgery, in particular the bilio-pancreatic diversion that was proven to allow diabetes remission through the normalization of insulin resistance [4]–[7]. After this operation as well as after Roux-en-Y gastric bypass, however, the biliary limb is excluded from food transit and it is no further explorable endoscopically to obtain mucosal biopsies. To harmonize the experimental design in humans and animals, we have thus chosen to demonstrate that the duodenum/jejunum of insulin resistant humans and mice secrete hormone/s inducing insulin resistance.

The natural evolution our study will be the isolation and identification of the IR hormone/s secreted by the duodenum-jejunum tracts. Their future identification might permit the development of new pharmacological agents for the treatment of type 2 diabetes. Insulin resistance is, in fact, a characteristic feature of type 2 diabetes and plays a central role in the pathogenesis of this disease although the presence of a concomitant β-cell failure is necessary. It is worldwide recognized that the skeletal muscle insulin resistance develops decades before β-cell failure [50]. The clinical implications of our study are related to the mechanisms of action of the jejunal hormone/s inducing IR which can explain the immediate metabolic and longer term effects of bariatric surgery in inducing the remission of type 2 diabetes [50].

## Supporting Information

Figure S1 **Effect of conditioned medium (CM) on ^308^Thr Akt phosphorylation in differentiated L6 myotubes.** *Panel A*: Effect of 25 µg/ml CM proteins on ^308^Thr Akt phosphorylation in differentiated L6 myotubes in the absence or presence of 100 nM insulin. Each bar is the mean of triplicate experiments with 4 different preparations of control and *db/db* CM and 1 preparation of Swiss CM. Data are normalized by β-actin amount and expressed as fold change versus control condition (control at 100 nM insulin set at 100). Data are mean ± s.d. ^#^ *P* = 0.08 vs. insulin stimulated ^308^Thr Akt phosphorylation in control. *Panel B*: Dose-response profile of insulin action on ^308^Thr Akt phosphorylation versus insulin concentration (nM) in logarithmic scale after 15 min co-incubation in the absence (squares) or presence (circles) of 25 µg/ml *db/db* CM proteins (^*^ *P*<0.05 and ^#^ *P* = 0.08 vs. control).
(EPS)

Appendix S1 L6 cells western blot analysis. Human skeletal muscle cell culture western blot analysis.
(DOCX)
