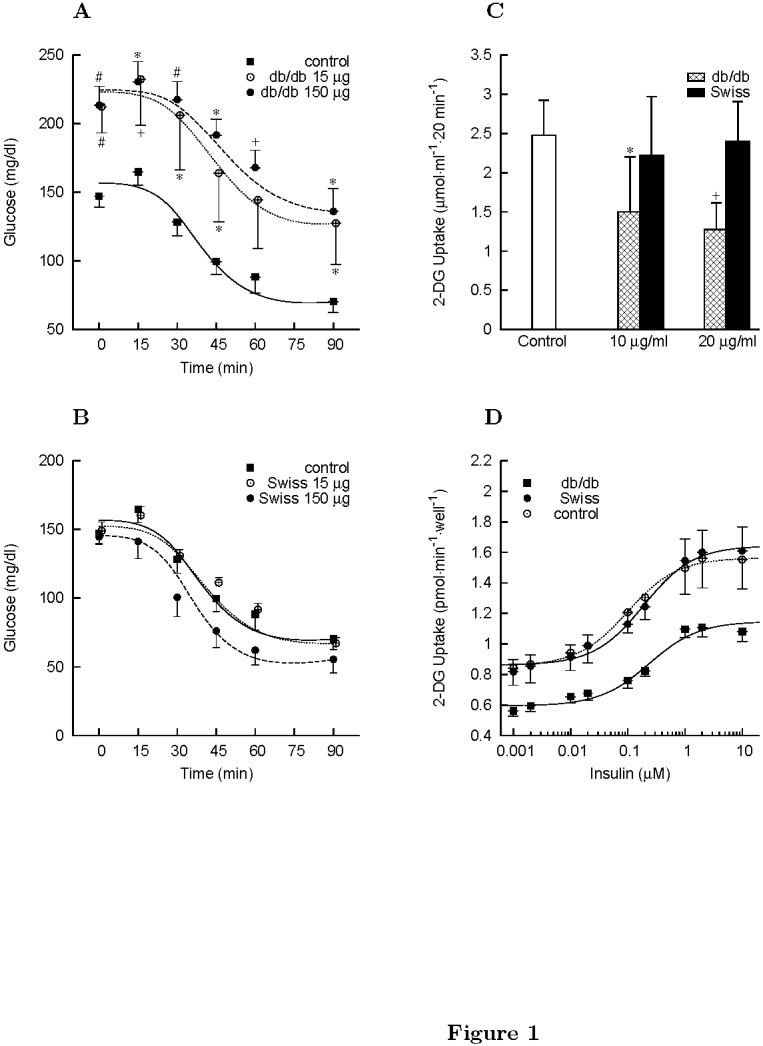# Correction: Jejunal Proteins Secreted by *db/db* Mice or Insulin-Resistant Humans Impair the Insulin Signaling and Determine Insulin Resistance

**DOI:** 10.1371/annotation/1a0f3773-d8d1-4cb4-ab18-649757f93139

**Published:** 2014-01-21

**Authors:** Serenella Salinari, Cyrille Debard, Alessandro Bertuzzi, Christine Durand, Paul Zimmet, Hubert Vidal, Geltrude Mingrone

There were a number of errors in the y and x axis labels for panels C and D of Figure 1 "Intraperitoneal insulin tolerance test on Swiss mice and effect of db/db and Swiss conditioned medium (CM) proteins on the in vitro glucose uptake." Please see the corrected Figure 1 here: 

**Figure:**