# Supplementary material for: Jejunal Proteins Secreted by db/db Mice or Insulin-Resistant Humans Impair the Insulin Signaling and Determine Insulin Resistance
Source: PLoS One. 2013 Feb 20;8(2):e56258. doi: 10.1371/journal.pone.0056258 (PMC3577828; doi:10.1371/journal.pone.0056258)
Supplement: Appendix S1 — L6 cells western blot analysis. Human skeletal muscle cell culture western blot analysis. (DOCX) [file pone.0056258.s002.docx]

***L6 cells western blot analysis***

L6 cells were harvested and lysed in ice-cold lysis buffer as previously described (21). All manipulations of cell lysates were performed at 4 °C. After centrifugation for 10 min at 12,000 x g, protein concentration in the supernatants was determined with Bratford assay using bovine serum albumin as standard.

Akt phosphorylations were detected using anti-phospho-Ser^473^ and anti-phospho-Thr^308^ antibodies (no. 9271 and no. 9275; Cell Signaling Technology, Danvers, MA, USA). Membranes were stripped and re-blotted with anti pan-Akt antibody (sc-1619; Santa Cruz Biotechnology, Santa Cruz, CA, USA). For the characterization of the other signaling pathways in L6 cells, we used anti-^9^Ser-GSK3β (no. 9336; Cell Signaling Technology) and anti-^389^Thr-p70 S6K1 (no. 9206; Cell Signaling Technology). In order to normalize for equal protein loading, membranes were stripped and re-blotted with an anti-actin antibody (n°A5060; Sigma-Aldrich).

***Human skeletal muscle cell culture western blot analysis***

Myotubes were homogenised at 4°C in 20 mmol/l Tris–HCl (pH 8.0), 138 mmol/l NaCl, 1% NP40 (*v*/*v*), 2.7 mmol/l KCl, 1 mmol/l MgCl_2_, 5% glycerol (*v*/*v*), 5 mmol/l EDTA, 1 mmol/l Na_3_VO_4_, 20 mmol/l NaF, 1 mmol/l dithiothreitol (DTT) and protease inhibitor cocktail. Lysates were centrifuged (12,000×*g*, 10 min) and stored at −80°C before use. 40 µg protein lysate was resolved on 10% SDS-PAGE (*w*/*v*). After transfer to polyvinylidenefluoride (PVDF) membranes, Akt phosphorylations were detected using anti-phospho-Ser^473^ (Cell Signaling Technology, Danvers, MA, USA). In order to normalize for equal protein loading, membranes were stripped and re-blotted with anti pan-Akt antibody (Santa Cruz Biotechnology, Santa Cruz, CA, USA). Antibodies anti ^9^Ser GSK3β were used to measure phosphorylated GSK3β (Cell Signaling Technology, Danvers, MA, USA).
